# A Cellular Senescence-Related Signature Predicts Cervical Cancer Patient Outcome and Immunotherapy Sensitivity

**DOI:** 10.21203/rs.3.rs-2769887/v1

**Published:** 2023-04-20

**Authors:** Huijing Shao, Xia Li, Pengfei Wu, Zixi Chen, Caihong Zhang, Hang Gu

**Affiliations:** The First Affiliated Hospital of Naval Medical University: Changhai Hospital; Huaian First People’s Hospital; fu dan da xue fu shu yi yuan fu chan ke yi yuan: Obstetrics and Gynecology Hospital of Fudan University; Putuo Hospital Affiliated to Shanghai University of Traditional Chinese Medicine: Shanghai PuTuo District Center Hospital; The First Affiliated Hospital of Naval Medical University: Changhai Hospital; The First Affiliated Hospital of Naval Medical University: Changhai Hospital

**Keywords:** cervical cancer, cellular senescence, bioinformatic analysis, prognosis, immunotherapy

## Abstract

Cervical cancer (CC) is among the most prevalent gynaecological malignancy. The rate of mortality and morbidity of patients with CC is high. Cellular senescence is involved in tumorigenesis as well as cancer progression. However, the involvement of cellular senescence in CC development is still unclear and requires further investigation. We retrieved data on cellular senescence-related genes (CSRGs) from the “CellAge” Database. We used TCGA-CESC and the CGCI-HTMCP-CC datasets as the training and validation sets, respectively. Eight CSRGs signatures based on the data extracted from these sets were constructed using “univariate” and “Least Absolute Shrinkage and Selection Operator Cox regression analyses”. Using this model, we calculated the risk scores of all patients in the training and validation cohort and categorised these patients into the low-risk group (LR-G) and the high-risk group (HR-G). Finally, compared to patients in the HR-G, CC patients in the LR-G demonstrated a more positive clinical prognosis; the expression of senescence-associated secretory phenotype (SASP) markers and immune cell infiltration was higher, and these patients had more active immune responses. In vitro studies showed increased SERPINE1 and IL-1α ((genes included in the signature) expression in CC cells and tissues. The eight-gene prognostic signatures could modulate the expression of SASP factors and the tumour immune micro-environment (TIME). It could be used as a reliable biomarker for predicting the patient’s prognosis and response to immunotherapy in CC.

## 1. Introduction

Globally, cervical cancer (CC) is the second most common gynaecological malignancy. In 2020, nearly 604,000 new incidences and 342,000 mortalities due to CC were reported [1]. According to previous reports, CC patients’ prognoses primarily depended on the disease stage during diagnosis. The survival rate of the early-stage patients is > 90%; however, the survival rate of CC patients with advanced and metastatic disease is < 20% [2]. Additionally, the therapeutic strategy also depends on the stage of the disease. The current treatment options for early-stage or localised CC include surgical resection, concurrent chemo, and radiotherapy. Systemic therapies are widely used for treating patients with metastatic CC [3–5]; however, their therapeutic efficacy is moderate. Immunotherapy is a rapidly evolving field, which involves modifying and recruiting the immune system to target cancer cells more efficiently and accurately. Currently, immunotherapy has been used in the treatment of various tumors. Common immunotherapy methods mainly include cytokine treatment, immune cell therapy, tumor vaccine, immune checkpoint inhibitors [6–8]. Patients with CC could also benefit from immunotherapy. Pembrolizumab (anti-PD-1) has been approved as an immunotherapy drug for treating patients with CC [9]. However, the efficacy of immunotherapy differs from patient to patient and is largely dependent on their immune status. Given the serious threat of cervical cancer to women’s health, attention should be paid to identify effective biomarkers which could facilitate early diagnosis, predict prognosis, and improve immunotherapeutic efficacy.

Cellular senescence is characterised by stable and terminal cell cycle arrest, accompanied by complex changes at the molecular level&. Research has demonstrated that cellular senescence has both a positive and negative impact on the development and advancement of cancer. Senescent cells undergo persistent cell cycle arrest, which helps maintain tissue homeostasis, prevent tumorigenesis by arresting cell proliferation and enhance immune surveillance of cancer cells [10–12]. However, the failure to eliminate senescent cells by the immune system and their accumulation in the tumour micro-environment (TME) could lead to adverse effects. Senescence-associated secretory phenotype (SASP) is a characteristic phenotype of cellular senescence, which includes multiple components like chemokines, cytokines, growth factors, interleukins, and proteases [13]. These SASP components could remodel tumor micro-environment (TME) and can promote tumorigenesis. Furthermore, the advent of high-throughput technology and several publicly available databases like “SeneQuest” [14], “CellAge” [15] and “Human Cellular Senescence Gene Database (HCSGD)” [16] could aid in identifying cellular senescence-associated gene signatures in cancer. Using senescence-associated genes, Luo et al. established a new model for predicting breast cancer patients’ survival outcomes [17]. However, the contribution of cellular senescence to the development of CC is still unknown and no risk models based on cellular senescence have been established for CC.

Therefore, in this study, we screened for cellular senescence-related biomarkers. In addition, we developed a risk model to predict the prognosis, TIME, and the response of patients to immunotherapy in CC. This risk model could aid clinicians in designing more effective therapeutic strategies.

## 2. Materials And Methods

### 2.1 Data Retrieval and Processing

The transcriptomic data of patients with CC were obtained from the “TCGA-TARGET-GTEx” database using the UCSC Xena (http://xena.ucsc.edu/) browser. This database was used for differential expression analysis. Further, the data on gene expression, as well as clinical and somatic mutations in patients with CC, were retrieved from “TCGA-CESC” (https://portal.gdc.cancer.gov/) dataset, which served as the training set. Additionally, the data on RNA sequencing and survival of patients with CC were retrieved from the “CGCI-HTMCP-CC” (https://portal.gdc.cancer.gov/) dataset, which served as the validation set. Samples without vital clinicopathological information or survival data were excluded from the study. Finally, we included 286 patients from “TCGA-CESC” dataset and 117 patients from the CGCI-HTMCP-CC dataset. We identified 279 genes from the “CellAge” (https://genomics.senescence.info/cells/) database as cellular senescence-related genes (CSRGs). Survival analysis of single gene was performed using GEPIA database (http://gepia.cancer-pku.cn/index.html).

### 2.2 Identification and Functional Enrichment of DEGs

Utilizing the “DESeq2” R package, we identified differentially expressed genes (DEGs) with a threshold criterion of “P < 0.05” and “|log2-fold change| ≥ 1”. Next, we employed the “clusterProfiler” R package [18] for performing “Gene Ontology (GO)” and “Kyoto Encyclopedia of Genes and Genomes (KEGG) pathway enrichment analyses” to determine the pathways that are significantly enriched by the DEGs (An adjusted P < 0.05). Finally, we constructed a volcano plot in the “ggplot2” R package [19] for visualising DEGs and the results of GO and KEGG analyses.

### 2.3 Establishing and validating the Prognostic Signature Associated with Cellular Senescence

We employed the “VennDiagram (version 1.7.3)” R package for constructing a Venn diagram to identify overlapping genes between the DEGs and CSRGs. First, the “univariate Cox regression” analysis was used to determine the correlation between the 23 overlapping genes and the survival outcomes of patients. Then, the “glmnet” R package [20] was employed to conduct the “least absolute shrinkage and selection operator (LASSO) Cox regression analysis” on significant genes (*P* < 0.05). Subsequently, we used 10-fold cross-validation in “LASSO Cox regression analysis” for constructing an optimal risk model. Finally, we used the risk model for calculating the risk scores based on the following formula:

1 $$\text{Riskscore}=\sum_{i=1}^{n}\left(\text{Coe }f_{i}*x_{i}\right)$$

The risk scores of all patients in the training set were calculated using this formula. Next, we used the median value as the parameter to categorise all patients into the high-risk group (HR-G) and the low-risk group (LR-G). Principal component analysis (PCA) was performed using the “prcomp” R package for subsequent clustering. Additionally, we performed Kaplan-Meier (KM) analysis using the “survival” and “survminer” R packages [21] to determine differences in the survival outcomes of the patient in the two groups. Finally, the ROC curves were generated using the “timeROC” R package to assess the predictive performance of the risk model. Next, we verified the predictive efficiency of the risk model in patients in the validation set using the abovementioned formula.

### 2.4 Clinical Significance of the Signature and a Novel Prognostic Nomogram

We employed the “Univariate and multivariate Cox regression analyses” for determining the independent clinical prognostic significance of the risk model. Next, the “rms (version 6.3.0)” R package was employed for constructing a predictive nomogram using the results of “univariate” and “multivariate Cox analyses”. First, the nomogram was constructed for predicting the 1-, 3- and 5-year overall survival (OS) rates of patients with CC. Subsequently, the calibration curves were plotted and “decision curve analysis (DCA)” was conducted to study the validity of the nomogram in the clinical setting.

### 2.5 Gene Set Enrichment Analysis

We identified DEGs in patients in the two risk groups using the “DEseq2” R package based on the following threshold criteria “*P* < 0.05” and “|log2-fold change| ≥ 1.” This was used to elucidate the mechanisms underlying CC pathogenesis. Subsequently, “gene set enrichment analysis (GSEA)” was performed on DEGs. “*P* < 0.05”, “false discovery rate q-value < 0.25”, and “normalised enrichment score > 1.5” were considered significantly enriched genes.

### 2.6 Evaluation of Immune Cell Infiltration and Tumour-Associated Gene Set Scores

We used the three most used immune analysis methods for assessing the infiltration of immunocytes in the TME of CC patients, (1) To determine differences in the TME of patients in both groups, we assessed the levels of stromal and immune cell infiltration using the “Estimation of STromal and Immune cells in MAlignant Tumor tissues using Expression data (ESTIMATE)” R package. (2) Next, we determined the proportion of 22 immune cell types using the “Cell-type Identification by Estimating Relative Subsets of RNA Transcripts (CIBERSORT)” R package. (3) Finally, we used “single-sample gene set enrichment analysis (ssGSEA)” for evaluating the percentage of 29 immunocytes and determining the enrichment scores of angiogenesis, epithelial-mesenchymal transition (EMT), and hypoxia-related genes. Angiogenesis-, EMT- and hypoxia-related gene sets were retrieved from the “Molecular Signatures database”. Table S1 shows the relevant marker genes.

### 2.7 Prediction of Response to Immunotherapy

We utilized the Wilcoxon test to comparethe immune checkpoint gene expression among patients in the two groups. The “IMvigor210 (http://research-pub.gene.com/IMvigor210CoreBiologies/)” dataset contains patients with metastatic urothelial cancer who were treated with an anti-PD-L1 agent. We used the “‘IMvigor210CoreBiologies” R package [22] on this data to determine the correlation between immunotherapeutic efficacy and the risk model. We used the median risk score to categorise these patients into two risk groups. Finally, the immunotherapeutic efficacy and clinical outcomes of patients in these two groups were compared.

### 2.8 Analysis of Mutations and Drug Sensitivity

The ‘maftools’ R package was used to convert original files from TCGA to the mutation annotation format for comparing mutational landscapes in patients in the two groups [23]. Finally, to examine the clinical response of patients to chemotherapy, the “pRRophetic” R package was used for calculating the half-maximal inhibitory concentration (IC_50_) values of 138 commonly used chemotherapy agents [24].

### 2.9 Sample Collection

We collected ten pair-matched CC and normal tissues from the Putuo Hospital Affiliated to Shanghai University of Traditional Chinese Medicine between May–December 2022 and stored them at −80°C. Next, we performed real-time polymerase chain reaction (qRT-PCR) on these samples. This study was approved by the Ethics Committee of Putuo Hospital Affiliated to Shanghai University of Traditional Chinese Medicine. Written informed consent was obtained from all participants.

### 2.10 Cell Culture

HcerEpic, CaSki, HeLa, and SiHa cells were purchased from the Shanghai Institute of Cell Biology (Shanghai, China). HcerEpic and CaSki cells were cultured in RPMI-1640 medium. HeLa and SiHa were cultured in DMEM, and α-MEM (Gibco), respectively. All mediums were supplemented with 10% FBS (Gibco), penicillin, and streptomycin. The cells were incubated at 5% CO_2_ and 37°C.

### 2.11 RNA Isolation, Complementary (cDNA) Synthesis, and qRT-PCR

We isolated total RNA using the Total RNA Extraction Reagent (Vazyme, Nanjing, China). RNA was reverse transcribed using a Vazyme reverse transcription kit to cDNA. We used the ChamQ SYBR qPCR Master Mix (Vazyme, Nanjing, China) to perform qRT-PCR. The internal loading control used for qRT-PCR was *GAPDH*. The sequences of primers: *SERPINE1*-forward primer: 5’-CCCACTTCTTCAGGCTGTT-3’; *SERPINE1*-reverse primer: 5’- GTGTGTCTTCACCCAGTCAT-3’; IL-1α-forward primer: 5’ CCCAAGATGAAGACCAACCA-3’; IL-1α-reverse primer: 5’ CCGTGAGTTTCCCAGAAGAA-3’.

### 2.12 Western Blotting

RIPA buffer (Epizyme, Shanghai, China) was used for cell lysis to extract total protein, which was separated using gel electrophoresis at 120 V and moved to PVDF membranes. After being blocked with 5% milk, the PVDF membranes were incubated with primary antibodies for an overnight period at 4°C, followed by secondary antibodies for an additional two hours at room temperature. An ECL system was used to visualize the protein bands (NCM Biotech, Suzhou, China). The protein bands were visualised using an ECL system (NCM Biotech, Suzhou, China). The primary antibodies used for western blotting were anti-SERPINE1 (66261–1-Ig, Proteintech, IL, USA) and anti-IL-1α (ab300501, Abcam) antibodies.

### 2.13 Statistical Analysis

We performed all statistical analyses with the help of the R (version 4.1.3) software and Perl. We compared the patient’s OS rates in both groups using KM analysis. The infiltration of immunocytes and the expression of immune checkpoint genes in patients from the two groups were compared using the Wilcoxon test, with a significance level of p < 0.05.

## 3. Results

### 3.1 Differentially Expressed CSRGs in patients with CC

**Figure 1:**
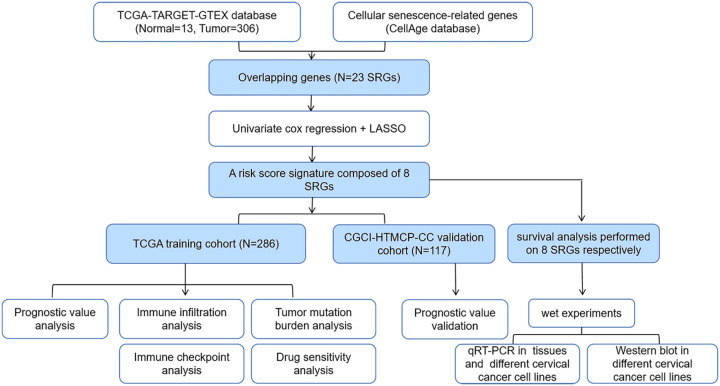
Study design and flow diagram.

**Figure 2:**
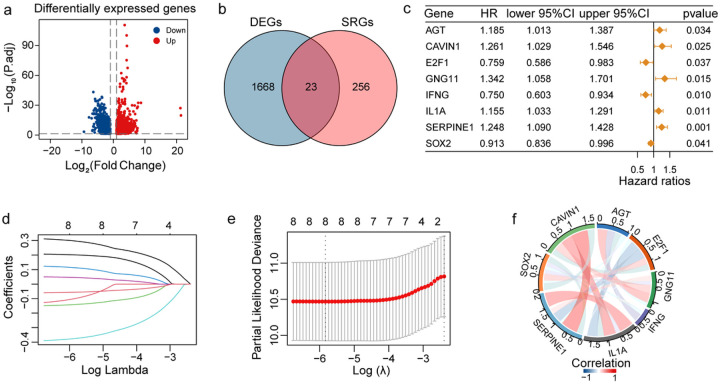
Identification of prognosis-related DEGs. (a) The volcano plot shows DEGs in tissues from CC and normal samples from TCGA-TARGET-GTEX dataset; red points indicate upregulation, and blue points indicate downregulation. (b) Venn plot shows 23 overlapping genes between DEGs and CSRGs. (c) The forest plot shows eight genes with P < 0.05 (univariate Cox regression analysis). (d) LASSO coefficient profiles of the eight prognosis-related genes. (e) Cross-validation for parameter selection. (f) Correlation network of the eight candidate genes.

Figure 1 shows the study design. We identified a total of 1691 DEGs in tissues of 306 patients with CC and 13 adjacent normal samples from TCGA-TARGET-GTEX dataset and visualised using a volcano plot. Of these DEGs, 695 were downregulated and 996 upregulated genes (Fig. 2a). GO and KEGG analysis revealed that the DEGs were associated with immune and inflammatory responses (Figure S1). To identify cellular senescence-related DEGs, we intersected DEGs with 279 CSRGs retrieved from the “CellAge” database. Finally, we identified 23 overlapping genes for further investigation (Fig. 2b).

### 3.2 Establishment of a CSRGs Prognosis Signature for CC

“Univariate Cox regression analysis” was performed on 23 overlapping genes to identify genes related to prognosis in CC. We performed the “LASSO COX regression analysis” on eight genes with “*P* < 0.05” (Fig. 2c and Table S1). We then identified eight significant prognosis-related genes using “LASSO COX regression analysis” with 10-fold cross-validation based on the optimal lambda value (Fig. 2d, 2e). We constructed the risk model based on the expression as well as coefficients of these eight genes. A correlation was observed among the expression levels of these eight prognostic genes. We can observe correlations among the eight prognostic genes, such as a positive correlation between *SERPINE1* and *IL-1α* expression and a negative correlation between *SERPINE1* and *E2F1* expression (Fig. 2f).

### 3.3 Assessing the Risk Model in Patients from the Training and Validation Sets

**Figure 3:**
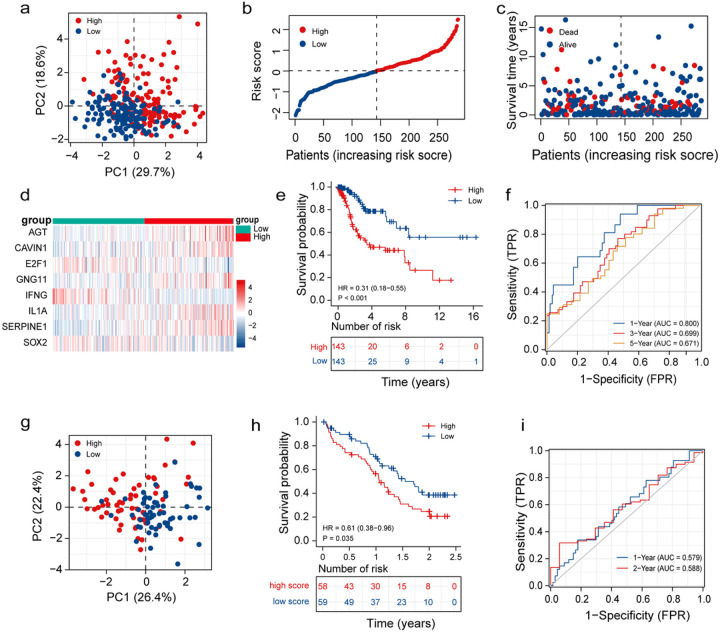
Establishment of a risk model based on CSRGs. (a) PCA plot of patients from TCGA dataset in the high- and LR-Gs. (b) Distribution of the risk scores of patients with CC from TCGA dataset. (c) The scatter plot shows the survival time and outcomes of patients with CC from TCGA dataset. (d) Heatmap of eight genes in the prognostic signature. (e) KM analysis in two groups. (f) Time-dependent ROC curves for predicting the 1-, 3-, and 5-year prognosis of patients. (g) PCA scatter plot of patients from the CGCI-HTMCP-CC dataset in the two risk groups. (h) KM analysis between the two risk groups in the CGCI-HTMCP-CC dataset. (i) Time-dependent ROC curves for predicting the 1- and 2-year prognosis of patients in the CGCI-HTMCP-CC dataset.

The median risk score served as the basis for distinguishing the patients from the training set into HR-G and LR-G. PCA revealed that the risk scores could efficiently distinguish patients in HR-G from patients in LR-G (Fig. 3a). Figure 3b shows a risk plot ranking and categorising patients into HR-G and LR-G. Interestingly, the number of deceased patients was higher in the HR-G (Fig. 3c). Furthermore, the heatmap in Fig. 3d displays the expression of the eight genes that constitute the risk model. High *AGT, CAVIN1, GNG11, IL-1α* and *SERPINE1* expressions were observed in patients in the HR-G. On the other hand, low *SOX2, E2F1*, and *IFNG* expressions were observed in patients in the LR-G. Additionally, KM survival curves showed that the prognosis of patients in the HR-G was poor compared to the LR-G (Fig. 3e). The “time-dependent ROC” analysis (Fig. 3f) showed that the AUC values (AUCs) for predicting the 1-year OS rate was 0.800, the 3-year was 0.6999, and the 5-year OS rate was 0.671. The results demonstrated the the risk model’s capability in predicting the prognosis of CC patients.To validate the reliability of the risk model in estimating patient’s prognoses, a risk score was calculated for each individual from the CGCI-HTMCP-CC dataset, and the entire cohort was divided into HR-G and LR-G. PCA showed significant differences in the two groups (Fig. 3g), thereby indicating that the risk signature could satisfactorily distinguish the prognoses of CC patients. Additionally, KM survival curves revealed that the survival rate of patients in the HR-G was poor (Fig. 3h). The AUCs for predicting the 1- and 2-year OS rates were 0.579 and 0.588, respectively (Fig. 3i). Together, these results suggest that the risk model could distinguish between the prognoses of patients.

### 3.4 Clinical Utility of the Eight CSRGs Signature and Development of a Novel Predictive Nomogram

**Figure 4:**
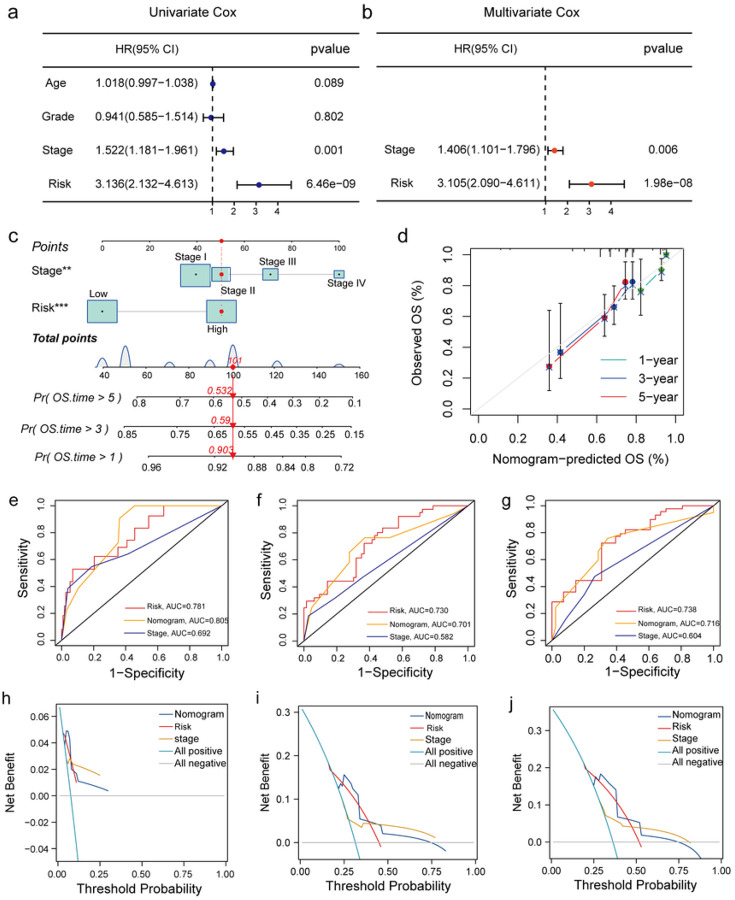
Clinical significance of the risk signature. (a) The forest plot shows univariate COX regression analysis results. (b) The forest plot shows multivariate COX regression analysis results of the prognostic signature and clinical variables. (c) Nomogram for assessing 1-, 3-, and 5-year OS rates. (d) The calibration curve for nomogram. (e-g) Time-dependent ROC curves of the nomogram, tumour stage, and the risk model for predicting the 1-, 3- and 5-year OS rates. (h-j) DCA of the clinical stage, the risk scores, and nomogram for predicting the 1-, 3- and 5-year OS rates.

We analysed the training set to identify if there was any correlation between the risk scores and the clinical parameters (age, grade, survival status, and tumour stage) of the patients. The risk scores of deceased (Fig. S2a) and elderly patients (Fig. S2b) were higher. However, no difference in the risk score was observed in patients with different tumour grades (Fig. S2c) or stages (Fig. S2d). Furthermore, “univariate COX regression analysis” revealed that the risk score of patients and the tumour stage were both reliable prognostic factors, which were subsequently included in the “multivariate COX regression analysis” (Fig. 4a). “Multivariate Cox regression” analysis showed that the risk score (*P* < 0.001) and tumour stage (*P* = 0.006) served as independent prognostic factors (Fig. 4b). Finally, we established a predictive nomogram by integrating the risk score and tumour stages of patients to determine the 1-, 3- and 5-year OS rates of patients with CC (Fig. 4c). The calibration curves demonstrated that the actual and the nomogram-predicted OS rates were highly similar (Fig. 4d). Furthermore, we calculated the AUCs of the risk model, nomogram, and tumour stage for determining the 1-, 3-, and 5-year OS rates using the “time-dependent ROC” analysis. Figure 4e–g revealed that the AUCs of the tumour stage, risk model, and nomogram were significant, thus indicating adequate predictive accuracy of these three factors. In addition, DCA revealed that the risk model, nomogram, and tumour stage demonstrated good performance in predicting the clinical net benefit (Fig. 4h–j).

### 3.5 Biological Processes Associated with the Risk Model and Correlation between SASP and the Risk Model

**Figure 5:**
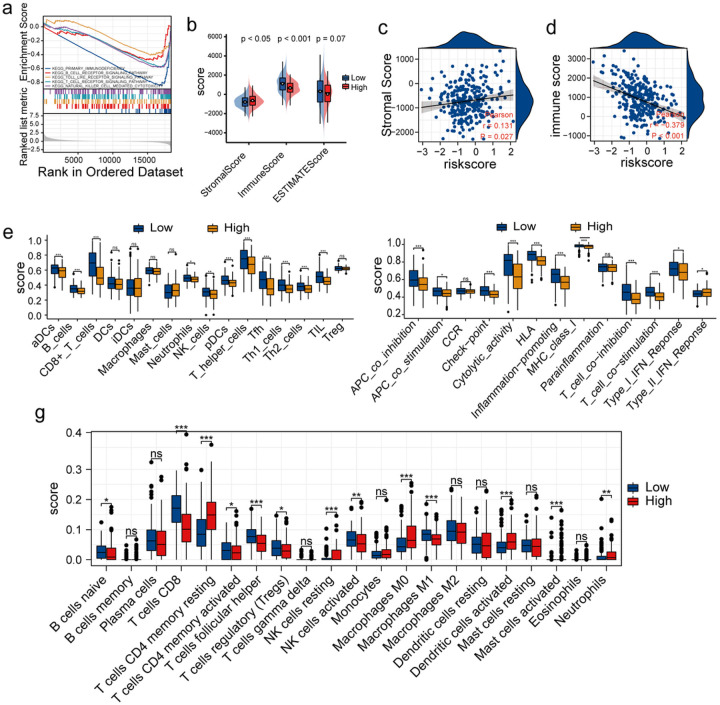
Correlation between the risk model and SASP, as well as the enrichment scores of specific gene sets. (a) The expression of SASP factors in patients in the HR-G and LR-Gs. (b) GSEA revealed activation of EMT, angiogenesis and hypoxia pathways in patients in the HR-G. (c-e) Comparison of the enrichment scores of EMT, angiogenesis and hypoxia-related genes in patients in the two risk groups (*, P < 0.05; **, P < 0.01; ***, P < 0.001; ns, no significant difference).

The results of the survival analysis of CC patients in our study confirmed the predictive efficiency of the risk model, prompting us to explore the underlying mechanisms. We identified 1058 DEGs in patients in the two groups in the training set (“*P* < 0.05” and “|log2-fold change| ≥ 1”). “GO and KEGG pathway enrichment analyses” revealed that multiple immune and inflammatory pathways altered (Fig. S3). Next, we compared the expression of different SASP factors in patients in the two groups. An increase in the expression of several SASP factors like chemokines (*CCL13, CCL26, CXCL1, CXCL5*, and *CXCL8*), interleukins (IL1A, IL1B, IL6, and IL7), growth factors and regulators (*VEGFA, IGFBP4, IGFBP6, IGFBP7*, and *NRG1*), proteases (*MMP1, MMP13, MMP14, PLAT, TIMP2*, and *SERPINE1*), and soluble receptors and ligands (*TNFRSF11B, TNFRSF1A*, and *PLAUR*) were observed patients in the HR-G (Fig. 5a). SASP factors promote tumour development by enhancing cell proliferation, angiogenesis, EMT, and chronic inflammation. GSEA revealed an increase in the activation of pathways associated with angiogenesis, EMT, and hypoxia in patients in the HR-G (Fig. 5b). ssGSEA showed significantly high enrichment scores of EMT, angiogenesis, and hypoxia-related genes in patients in the HR-G (Fig. 5c–e). These results indicate that high-risk patients had a more malignant phenotype, and their prognosis was poor from a molecular perspective.

### 3.6 Relationship between the Signature and the Immune Landscape

**Figure 6:**
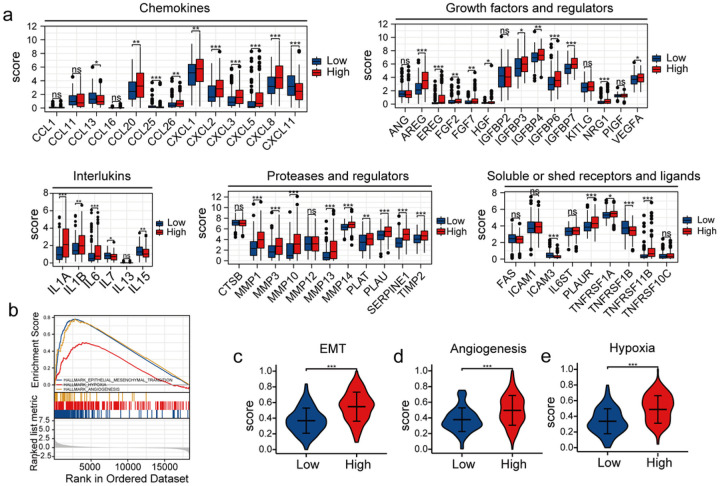
Evaluation of immune cell infiltration. (a) Multiple significant immune-related related pathways were identified using GSEA. (b) Differences in the immune and stromal scores of patients in the HR-G and LR-G. (c) Stromal scores of patients in the two groups. (d) Immune scores of patients in the two groups. (e) Infiltration of 16 immune cell types was measured using the “ssGSEA” algorithm. (f) Enrichment scores of 13 immune-related functions were determined using the “ssGSEA” algorithm. (g) The abundance of 22 immune cells in patients in the HR-G and LR-G were determined using the “CIBERSORT” algorithm (*, P < 0.05; **, P < 0.01; ***, P < 0.001; ns, no significant difference).

SASP is characterised by the ability to induce inflammation. Given the proinflammatory nature of SASP, it is likely that senescent cells can attract immune cells. Figure 6a shows a significant enrichment of multiple pathways related to immune responses like natural killer cell-mediated cytotoxicity, the B, T cells, and Toll-like receptor signalling pathways in patients in the LR-G. These results suggest that the risk model could aid in predicting the immune status of patients with CC. Subsequently, we used algorithms like the “ESTIMATE”, “CIBERSORT”, and “ssGSEA” to assess the levels of immune cell infiltration in TME. The “ESTIMATE” algorithm was used to perform immune analysis in patients in the LR-G. In the LR-G, CC patients had higher immune scores and lower stromal scores than in the HR-G. (Fig. 6b). Similarly, Pearson correlation analysis showed a positive correlation between the patient’s stromal and risk scores (r = 0.131, *P* = 0.027; Fig. 6c). Further, a negative correlation between the immune and risk scores was observed (r = −0.379, *P* = 0.001; Fig. 6d). Additionally, ssGSEA showed significant differences between the two groups in terms of immune profiles. A significant increase in B cells, neutrophils, NK cells, CD8 + T cells, and Th and Th2 cell infiltration was observed in patients in the LR-G (Fig. 6e). Additionally, multiple immune function signatures were significantly activated in patients in the LR-G (Fig. 6f). Immune analysis performed using the “CIBERSORT” algorithm revealed an increase in the abundance of activated NK cells, naïve B cells, neutrophils, T follicular helper, M1 macrophages and CD8 + T cells, as well as activated mast cells in patients in the LR-G. However, a significant increase in the abundance of neutrophils, resting memory CD4 + T cells, and M0 macrophages was observed in patients in the HR-G (Fig. 6g). Together, these results showed increased antitumour immune activity in low-risk patients. Furthermore, these findings indicate that the risk model was an effective tool in assessing the immune status of CC patients with different degrees of risk.

### 3.7 Correlation between the Risk Model and Immune Checkpoints as well as its Potential to Predict Immunotherapy Response

**Figure 7:**
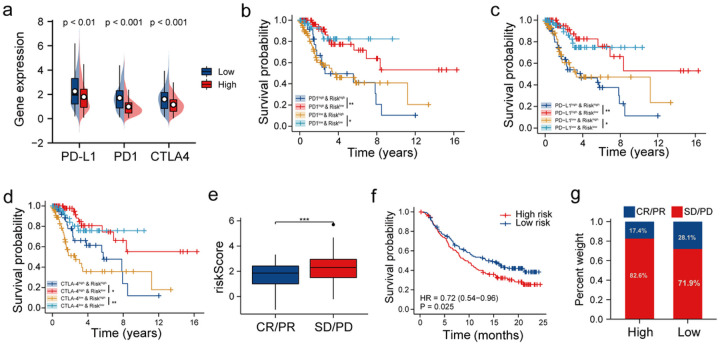
Efficiency of the risk signature in predicting response to immunotherapy. (a) Expression of immune checkpoint genes like PD1, PD-L1 and CTLA-4 in patients in both groups. (b–d) KM survival curves for assessing the survival of patients from TCGA dataset stratified based on the risk scores and the expression of immune checkpoint genes like PD1, PD-L1 and CTLA-4. (e) The risk scores of patients with different clinical responses (CR/PR, complete response/partial response; SD/PD, stable disease/progressive disease) (f) KM survival curves of patients from the IMvigor210 cohort in the two risk groups. (g) Comparison of the clinical response rates for anti-PD-L1 immunotherapy.

Few studies have shown the significant involvement of immune checkpoint genes in regulating the infiltration of immune cells. Our results demonstrated that a correlation exists between the risk model and the tumour immune micro-environment of patients with CC. Therefore, we determined *PD1*, *CTLA-4*, and *PD-L1* expression in patients in the two groups to understand the complex correlation among the risk scores, immunocytes infiltration, and the expression of immune checkpoint genes. Figure 7a shows a significant increase in *PD1, CTLA-4*, and *PD-L1* expression in patients in the LR-G compared to the HR-G. Next, we performed survival analysis on patients divided into four groups based on the signature and *CTLA-4, PD1*, and *PD-L1* expression. The survival of patients expressing high *PD1* levels in the LR-Gs was significantly longer compared to patients expressing high *PD1* levels in the HR-G (*P* = 0.006; Fig. 7b). Additionally, the OS of patients expressing low *PD1* levels in the LR-G was significantly longer (*P* = 0.038; Fig. 7b). Figures 7c and 7d show the survival pattern of patients classified in groups based on the risk scores, *PD-L1* and *CTLA-4* expressions in the training set were similar. Furthermore, we assessed the ability of the risk model to predict the efficacy of immunotherapy in the IMvigor210 cohort. The response (*P* < 0.001; Fig. 7e) and OS (*P* = 0.025; Fig. 7f) of patients with low-risk scores to anti-PD-L1 drugs were significantly better compared to patients in the HR-G. Further, in the LR-G, the response of patients (28.1%) to anti-PD-L1 therapy was better compared to the HR-G (17.4%; Fig. 7g). Results suggest that those with low-risk CC may gain more benefit from anti-PD-L1 treatment.

### 3.8 Correlation between the Risk model, Tumour Mutational Burden (TMB), and Drug Sensitivity

**Figure 8:**
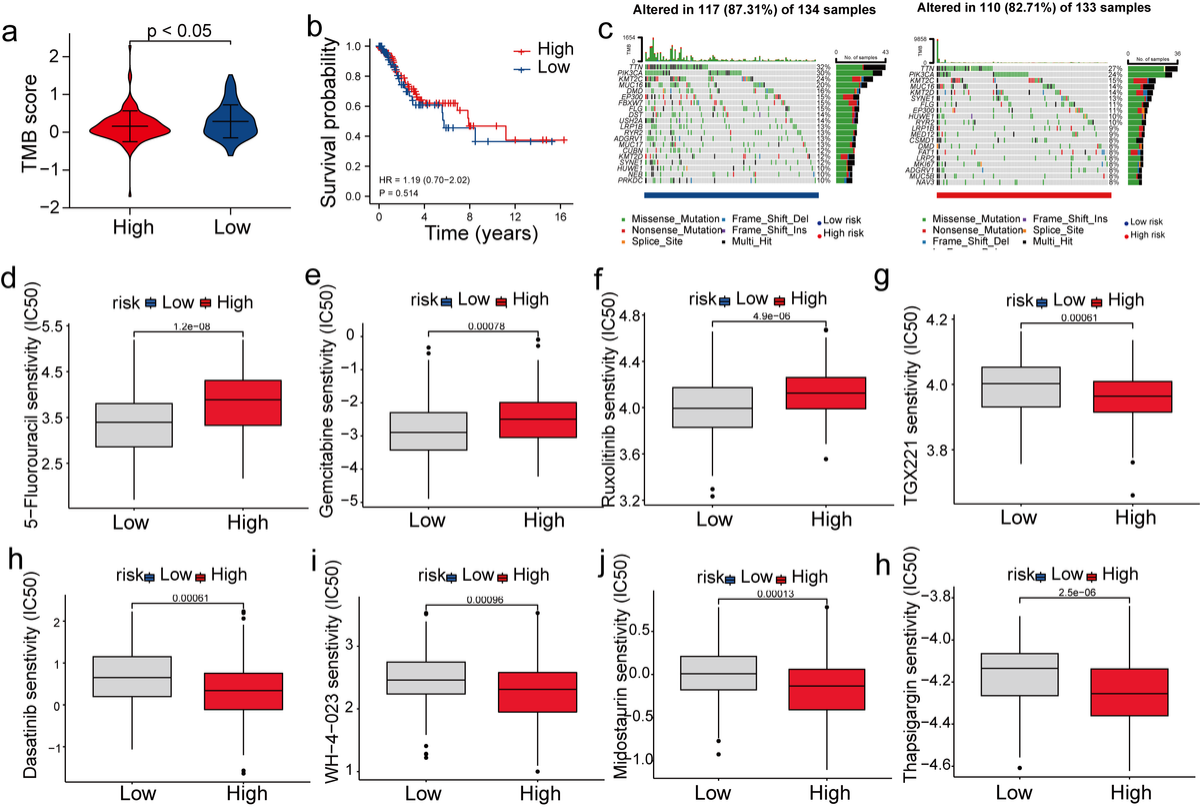
Correlation between the risk score, TMB and chemosensitivity. (a) Comparison of TMB scores between the two risk groups. (b) KM analysis in the two risk groups. (c) The waterfall plot shows somatic mutations in the two risk groups. (d-h) Correlation between the risk scores and sensitivity to various chemotherapy drugs.

TMB is a reliable factor for evaluating the immune response against tumours. The results showed that the TMB scores of patients in the LR-G were higher compared to the HR-G, thus indicating that low-risk patients could benefit more from immunotherapy (Fig. 8a). Next, to assess the impact of TMB on clinical outcomes, we compared the survival rates of patients in the low- and high-TMB groups, and found no significant difference between the two (Fig. 8b). Additionally, we examined the frequency of somatic mutations in both groups. Figure 8c shows TOP20 driver genes with the most mutation and the somatic mutation profiles. In both groups, the most frequently mutated genes were *MUC16, PIK3CA, TTN*, and *KMT2C*; however, the frequency of mutation of these genes was higher in patients in the LR-G compared to the HR-Gs. Currently, resistance to chemotherapy is a serious problem in cancer therapeutics. Therefore, we examined the therapeutic efficacy of 138 chemotherapeutic drugs in patients from TCGA dataset. The responses of patients in the LR-G to 38 drugs, including 5-fluorouracil, gemcitabine, and ruxolitinib, were positive (Fig. 8d–f). In the HR-G, the response of patients to dasatinib, thapsigargin, WH-4–023, midostaurin, and TGX22 was positive (Fig. 8g, 8h).

### 3.9 Expression of Signature Genes in CC Cells and Tissues

**Figure 9:**
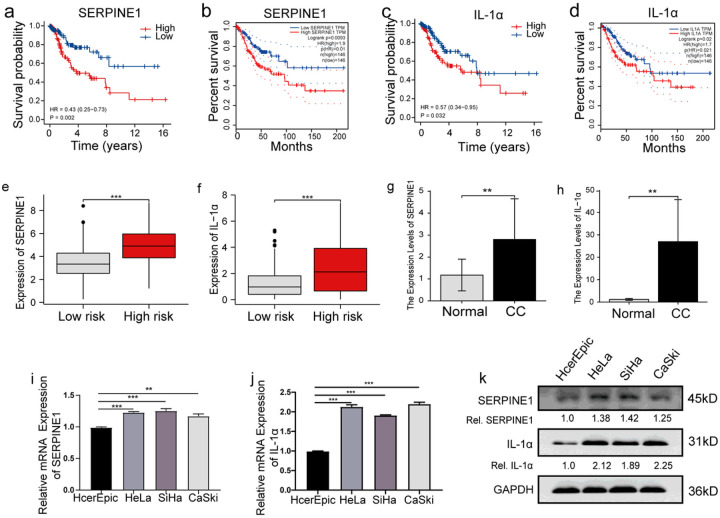
SERPINE1 and IL-1α expression in CC cells and tissues (a, c) KM survival curves to determine the survival of patients categorised based on SERPINE1 and IL-1α expression. (b, d) KM survival curves to determine the survival of patients from the GEPIA database categorised based on SERPINE1 and IL-1α expression. (e, f) Relative SERPINE1 and IL-1α mRNA expression in patients in the two risk groups. (g, h) Relative SERPINE1 and IL-1α mRNA expression in normal and CC tissues. (i-k) Relative SERPINE1 and IL-1α mRNA and protein expression in normal cervical epithelial cells and CC cells.

The risk model comprised eight CSRGs. The survival of patients with CC expressing high *IL-1α* and *SERPINE1* levels was poor (Fig. 9a, 9c). These results were verified using data from the GEPIA database (Fig. 9b, 9d). In addition, an increase in *SERPINE1* and *IL-1α* expression was observed in patients in the HR-G (Fig. 9e, 9f). These results suggest a close correlation between *SERPINE1* and *IL-1α* expression and the occurrence as well as the development of CC. Therefore, we determined *SERPINE1* and *IL-1α* expression in ten paired normal and CC tissues. An increase in *SERPINE1* and *IL-1α* expression was observed in CC tissues compared to normal tissues (Fig. 9g, 9h). Additionally, compared to normal cervical epithelial cells, an increase in SERPINE1 and IL-1α expression was observed in CC cells (Fig. 9i, 9j) at mRNA as well as protein levels (Fig. 9k).

## 4. Discussion

Cellular senescence is a stable cell cycle arrest that remodels the TME through various cancer hallmarks, such as tumour proliferation, migration, invasion, angiogenesis, EMT, and tumour immune response, thereby affecting cancer patients’ prognosis [25, 26]. However, the correlation between cellular senescence and CC is still unclear. In this study, we sought to explore the potential role of cellular senescence in the pathogenesis of CC using bioinformatics and in vitro experiments to better predict the prognosis of CC and provide novel targets for its treatment.

Firstly, we explored the expression of senescence-associated genes in normal and CC samples using databases. We found a total of 23 senescence associated genes that were differentially expressed in CC samples. These 23 genes include *AGT, SOX2, E2F1, GNG11, SERPINE1, IL-1α, IFNG, CAVIN1, CDKN2B, CXCL1, HEPACAM, HSPB2, ID1, IGFBP5, ING2, KL, MMP9, PMVK, SFN, SIX1, TNFSF15, VENTX, ZFP36*. This result preliminarily illustrated the important role of cellular senescence in the disease occurrence and progression of CC.

With the development of high-throughput sequencing technology as well as computer algorithms, constructing gene sets for disease prediction has provided strong support for prognostic prediction of tumors, which is beneficial for optimizing treatment decisions in clinical practice. Numerous gene signatures have been developed for tumor prognosis prediction, including cell senescence gene sets. For example, a study has established four senescence-related genes (*BAK1, DKK1, CDKN2A*, and *MIF*) based prognosis model for predicting the patient’s survival rate in head and neck squamous cell carcinoma [27]. Lin et al. [28] identified three cellular senescence clusters associated with different patient prognoses by analysing 278 CSRGs in lung adenocarcinoma. However, cellular senescence-related gene sets associated with patient prognoses have not been identified and validated in CCs. Therefore, we established a risk model consisting of eight CSRGs like *AGT, SOX2, E2F1, GNG11, SERPINE1, IL-1α, IFNG*, and *CAVIN1*. We used the median risk score as a parameter to divide the CC patients into the HR-G and LR-G. The survival and Cox regression analyses revealed that the risk signature could independently predict patients’ survival outcomes in CC. Additionally, a nomogram was established by integrating the risk scores and tumour stage. This nomogram verified the predictive efficiency and clinical utility of the risk model. Collectively, these results demonstrate that our risk model is capable of predicting the patient’s prognosis, which would aid in identifying biological factors involved in CC development.

SASP is an important feature of cell senescence, which includes cytokines, chemokines, growth factors, and proteases. Different SASP molecules serve different functions in the TME. We therefore analyzed the expression of SASP molecules in the HR-G and LR-G, and we found that multiple SASP molecules, including IL-6, IL-8, IL-1β, and VEGFA, were increased in high-risk CC patients. This is consistent with earlier findings. Multiple studies have shown that IL-6 is important for cervical carcinogenesis. Pan et al. conducted an immunohistochemistry analysis of IL-6 expression in CC tissues, discovering a significantly elevated expression in tumor tissues [29]. Additionally, research has shown that IL-6 is abundantly expressed in invasive CC and is implicated in the pathogenesis of HPV-related CC [30]. All the above findings suggest that IL-6 is a detrimental factor for the development of CC. IL-8 is a pro-inflammatory factor that promotes tumor growth. Fujimoto et al. [31] found that CC patients with high levels of IL-8 had an extremely poor prognosis, while patients with lower levels had a better 24-month survival rate, which indicates IL-8 is a prognostic indicator of CC. In 2017, Jia et al. [32] found that IL-8 were associated with the tumorigenesis of CC, and exogenous IL-8 promotes the carcinogenic potential of HeLa cells. VEGFA is a key factor in blood vessel formation, and previous studies have shown that the expression amount of serum VEGFA is upregulated in CC, and targeting VEGFA is beneficial for the treatment of CC [33, 34]. It therefore has the potential to be an effective treatment modality for cervical cancer by modulating SASP molecules in the TME.

Another important finding in our study is the significant correlation between CSRGs and the composition of tumour-infiltrating immune cells. Many studies have shown that cellular senescence is associated with the TIME [35]. Senescent cells secrete numerous cytokines and chemokines to induce immune cells and promote the body’s immune response. Multiple studies have confirmed that most of cervical cancer is HPV positive, and the body shows antiviral immune response after infection with HPV virus [35]. Therefore, the TIME is important for the development of cervical cancer, which deserves to be fully studied. In this work, GSEA analysis revealed the up-regulation of several pathways associated with immune responses like natural killer cell-mediated cytotoxicity, the B, T, and Toll-like receptor signalling pathways in patients in the LR-G. Our correlation analysis revealed that the stromal score had a positive correlation with the risk score, while the immune score had a negative correlation with the risk score. Based on this, we analysed the composition of immunocytes, and we found that most immune cells were up-regulated in the LR-G, including B cells, CD8 + T cells, NK cells, and neutrophils. These results illustrate a more active and complex immune response in patients in the LR-G, which also lays the foundation for immunotherapy in CC.

Immunotherapy is a rapidly developing therapeutic strategy which holds tremendous potential in clinical settings. Immunotherapy targets and eliminates tumour cells by activating the immune system of the patient. Studies have shown the efficacy of immunotherapy in treating various solid cancers like lung, breast, and renal [36–39]. Additionally, studies have demonstrated the benefits of immunosuppressive agents targeting PD-1 and CTLA-4 or its primary ligand PD-L1 in treating patients with advanced and metastatic CC [40, 41]. Cellular senescence-related immune remodelling could influence the efficacy of immune checkpoint blockade. Our results showed an increase in PD-1, CTLA-4, and PD-L1 expression in patients in the LR-G, thus indicating a higher sensitivity of these patients to immune checkpoint blockade therapy. Additionally, in the IMvigor210 cohort, patients with low-risk scores were highly sensitive to PD-L1 inhibitors. Therefore, the risk model could aid in screening patients who could benefit from combination therapy.

We established the risk model based on eight CSRGs. KM analysis showed an independent association between *SERPINE1* and *IL-1α* expression and the prognosis of patients with CC. SERPINE1 negatively regulates the pericellular proteolytic pathway. Studies have shown a correlation between high *SERPINE1* expression and poor disease as well as shorter disease-free survival outcomes in several cancers, like, breast and gastric [42, 43]. Hazelbag et al. used “multivariate COX regression analysis” and identified *SERPINE1* as a strong independent prognostic factor for CC. Additionally, the study has shown an association between *SERPINE1*, poor survival, and disease recurrence in a subgroup of patients with CC without lymph node metastasis [44]. Interestingly, our results showed high *SERPINE1* expression in patients in the HR-G. The clinical outcome of CC patients expressing high *SERPINE1* levels was poor. In addition, *SERPINE1* expression was higher in CC cells and tissues. IL-1α is a crucial cytokine involved in inflammatory processes and promotes cancer pathogenesis. However, IL-1α exert pro- and anti-cancer effects; hence its involvement in cancer progression is still controversial. Liu, S. et al. [45] showed that IL-1α promotes breast cancer progression by increasing the activation of the NF-kB and STAT3 signalling pathways. Interestingly, Dagenais, M. et al. [46] showed that IL-1α suppresses breast cancer by inhibiting cell proliferation via the IL-1α signalling pathway. However, no study has reported IL-1α expression and functions in CC. Our results showed an increase in IL-1α expression in patients in the HR-G. The prognosis of patients expressing high IL-1α levels was poor. Additionally, an increase in IL-1α expression was observed in the CC cells and tissues, thus indicating that IL-1α could promote malignant transformation of cells, thereby detrimental to the prognosis of patients with CC. In vitro as well as in vivo should be conducted to determine the involvement of SERPINE1 and IL-1α in CC.

However, our study has several limitations. First, we used data extracted from publicly available databases. Hence, prospective studies involving human subjects are required to validate our results. Additionally, cell-based and animal experiments should be performed to enhance our understanding of the mechanisms of CSRGs in the progression of CC.

## 5. Conclusion

We constructed a risk model comprising eight CSRGs to predict the prognosis of patients with CC. In addition, we used the risk model for determining the clinical outcomes and immune cell infiltration profiles of patients with CC. Finally, our risk model may help to design accurate and personalised therapeutic strategies for patients with CC.

## Data Availability

The datasets analyzed in this study are available from TCGA-TARGET-GTEX datasets in UCSC Xena (http://xena.ucsc.edu/) and TCGA official website (https://portal.gdc.cancer.gov/repository).
